# Characterization of Orange Peel Extract and Its Potential Protective Effect against Aluminum Chloride-Induced Alzheimer’s Disease

**DOI:** 10.3390/ph16010012

**Published:** 2022-12-22

**Authors:** Nourhan Mohammad Abd El-Aziz, Mohamed Gamal Shehata, Tawfiq Alsulami, Ahmed Noah Badr, Marwa Ramadan Elbakatoshy, Hatem Salama Ali, Sobhy Ahmed El-Sohaimy

**Affiliations:** 1 Department of Food Technology, Arid Lands Cultivation Research Institute (ALCRI), City of Scientific Research and Technological Applications (SRTA-City), Alexandria 21934, Egypt; 2 Food Research Section, R&D Division, Abu Dhabi Agriculture and Food Safety Authority (ADAFSA), Abu Dhabi P.O. Box 52150, United Arab Emirates; 3 Department of Food Science & Nutrition, College of Food and Agricultural Sciences, King Saud University, Riyadh 11451, Saudi Arabia; 4 Food Toxicology and Contaminants Department, National Research Centre, Dokki, Cairo 12622, Egypt; 5 Food Science Department, National Research Centre, Dokki, Cairo 12622, Egypt; 6 Department of Technology and Organization of Public Catering, Institute of Sport, Tourism and Service, South Ural State University (SUSU), 454080 Chelyabinsk, Russia

**Keywords:** natural products, orange peels, Alzheimer’s disease, anti-acetylcholinesterase, docking, in silico

## Abstract

Alzheimer’s disease (AD) is a devastating neurodegenerative disorder without a cure. Hence, developing an effective treatment or protective agent is crucial for public health. The present study aims to characterize orange peel extract (OPE) through in vitro and in silico studies. Furthermore, it examines the protective effect of OPE against experimentally-induced Alzheimer’s disease in rats. The total phenolic and flavonoid content of OPE was 255.86 ± 1.77 and 52.06 ± 1.74 (mg/100 g), respectively. Gallic acid, the common polyphenol in OPE detected by HPLC was 3388.60 μg/100 g. OPE antioxidant IC_50_ was 67.90 ± 1.05, 60.48 ± 0.91, and 63.70 ± 0.30 by DPPH, ABTS and Hydroxyl radical scavenging activity methods, respectively. In vitro anti-acetylcholinesterase (AChE) IC_50_ was 0.87 ± 0.025 mg/mL for OPE and 2.45 ± 0.001 mg/mL for gallic acid. Molecular docking analysis for human AChE (4EY7) with donepezil, gallic acid, and acetylcholine showed binding energy ΔG values of −9.47, −3.72, and −5.69 Kcal/mol, respectively. Aluminum chloride injection (70 mg/Kg/day for 6 weeks) induced Alzheimer’s-like disease in male rats. OPE (100 and 200 mg/kg/d) and gallic acid (50 mg/kg/d) were administered orally to experimental animals for 6 weeks in addition to aluminum chloride injection (as protective). OPE was found to protect against aluminum chloride-induced neuronal damage by decreasing both gene expression and activity of acetylcholinesterase (AChE) and a decrease in amyloid beta (Aβ42) protein level, thiobarbituric acid-reactive substances (TBARS), and nitric oxide (NO), and increased reduced glutathione (GSH) level and activity of the antioxidant enzymes in the brain tissues. Additionally, gene expressions for amyloid precursor protein (APP) and beta secretase enzyme (BACE1) were downregulated, whereas those for presinilin-2 (PSEN2) and beta cell lymphoma-2 (BCL2) were upregulated. Furthermore, the reverse of mitochondrial alternation and restored brain ultrastructure might underlie neuronal dysfunction in AD. In conclusion, our exploration of the neuroprotective effect of OPE in vivo reveals that OPE may be helpful in ameliorating brain oxidative stress, hence protecting from Alzheimer’s disease progression.

## 1. Introduction

Dementia caused by Alzheimer’s disease (AD) is a complex illness without a treatment [1]. The prevalence of Alzheimer’s disease (AD) is rising as the number of elderly people increases [2]. Currently, more than 55 million people live with dementia worldwide, and there are nearly 10 million new cases every year. Dementia results from a variety of diseases and injuries that primarily or secondarily affect the brain. Alzheimer’s disease is the most common form of dementia and may contribute to 60–70% of cases [3].

Protein amyloid beta (plaques) and tau (tangles) accumulate outside and inside of neurons, respectively, as characteristic pathologies of Alzheimer’s disease [4]. These alterations are associated with neuronal death and brain tissue damage [5]. Several studies have found that mitochondrial dysfunction is highly prevalent in AD, pointing to a crucial role for mitochondrial homeostasis in the progression of this disease [6]. It is well-reported that several pathological conditions of AD, including Aβ pathology, disturb mitochondrial dynamics via direct or indirect processes [7].

The majority of the metals found in the crust of the Earth are aluminum. It’s in everything we consume: food, water, dust, air, beverages, flavored drinks, energy drinks, and medicine [8]. Due to its extremely high affinity to transferrin receptors, aluminum is able to cross the blood–brain barrier (BBB) and accumulate in the brain [9]. Kawahara states that this metal causes neuronal death both in vivo and in vitro [10]. Through its ability to cross-link hyperphosphorylated proteins, aluminum may actively contribute to the etiology of major neuropathologic lesions in Alzheimer’s disease and similar illnesses [11].

Six medications for the treatment of Alzheimer’s disease have been greenlighted by the Food and Drug Administration (FDA) in the United States. There are now five medications that temporarily treat Alzheimer’s symptoms but do not change the underlying brain changes of Alzheimer’s or alter the course of the disease. These medications include donepezil, rivastigmine, galantamine, memantine, and memantine coupled with donepezil [3].

With the exception of memantine, these medications alleviate symptoms by boosting levels of neurotransmitters. Memantine prevents glutamate, a neurotransmitter that can overstimulate and even destroy neurons, from doing so in the brain. The potential adverse effects of these five medications are generally minor and may include side effects such as headaches and nausea [3]. As of June 2021, aducanumab, the sixth medicine on the list, is the first drug approved by the FDA to treat the underlying biology of Alzheimer’s disease rather than its symptoms. Beta-amyloid plaques in the brain are diminished as a result. However, not everyone with Alzheimer’s should try this treatment, as it is not a cure. Amyloid-related imaging abnormalities (ARIA) are a sign of brain swelling and are more common with aducanumab than with other medications approved to treat Alzheimer’s disease [12]. These synthetic drugs are associated with a number of side effects, possess much economic burden and are not implemented for all cases of AD [13]. Therefore, there is a high demand for discovering new remedies of natural origin obtained from medicinal plants to be directed for protection from, slowing down or halting of AD disease progression in its early stages. The protective effect of polyphenols against Alzheimer’s disease was recently reviewed by Chiara Colizzi [14]. In addition, research has shown that phenolic compounds derived from plants and fruits have a variety of beneficial biological effects, such as antioxidant, antimicrobial, anti-inflammatory, anticancer, and hepato-protective properties [15,16,17].

Aqueous orange peel extract had many food applications such as supplementation in minced beef to suppress lipid oxidation [18] and milk for increasing the antioxidant activity and total polyphenol content, and decreasing the total microbial count [19].

Peels tend to be the predominant byproduct of citrus juice production, contributing to environmental contamination [19]. Over 100 million tons of citrus fruit is harvested every year, making it the world’s most prolific fruit crop. It is estimated that typical food processing processes squander 20% of the entire weight of citrus peels as by-products [20]. Orange peels are sources of various bioactive ingredients, such as phenolics, flavonoids, tannins, and limonoids especially, that are unique to other plants [21]. Antimicrobial, antioxidant, and anti-cancer properties are just a few of the many biological functions exhibited by these bioactive chemicals [22]. Therefore, citrus by-products applications should be studied in order to turn them into value-added products [23]. In this study, in vitro and computer simulations are conducted to learn more about orange peel extract (OPE). Moreover, we assess a number of biochemical, molecular, and brain ultrastructural biomarkers to investigate OPE’s protective impact against a rat model of Alzheimer’s disease induced by aluminum chloride injection (70 mg/Kg/day for 6 weeks).

## 2. Results

### 2.1. Phytochemical and HPLC Analysis

Total phenolics (TP) and flavonoids (TF) concentrations were 255.86 ± 1.77 and 52.06 ± 1.74 (mg/100 g), respectively (Table 1). Twelve polyphenolic compounds were detected by HPLC in OPE (Table 2). The most abundant polyphenol was gallic acid with 3388.60 μg/100 g concentration. Moreover, rutin, naringenin, ferulic acid, and quercetin was detected with concentrations 1952.64, 1967.43, 1335.96, and 1266.97 μg/100 g, respectively.

### 2.2. Biochemical Properties of OPE

#### 2.2.1. Antioxidant Activity

The antioxidant activities of OPE was evaluated using DPPH, ABTS and Hydroxyl radical scavenging activity and the results in IC_50_ were 67.90 ± 1.05, 60.48 ± 0.91, and 63.70 ± 0.30 μg/mL, respectively (Table 1).

#### 2.2.2. Anti-Acetylcholinesterase Power

The effectiveness of OPE and gallic acid in inhibiting AChE, an enzyme that is hyperactivated in AD, was investigated in vitro. Critical concentrations for inhibition (IC50) of OPE, gallic acid, and donepezil were 0.87 ± 0.025, 2.45± 0.001, and 0.0034 ± 0.0 mg/mL, respectively (Table 1).

### 2.3. Computational Docking Analysis

**Figure 1:**
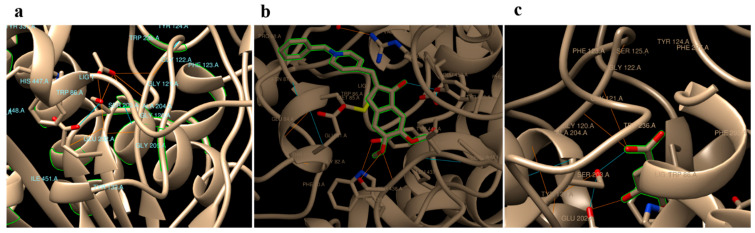
Predicted binding modes of acetylcholine (**a**), Aricept (**b**), and gallic (**c**) with hAChE (PDB Code: 4EY7).

*In silico* docking analyses of gallic acid, donepezil and native substrate (acetylcholine) separately against Homo sapiens AChE (PDB code: 4EY7) revealed hydrogen binding energy (ΔG) values −3.72, −9.47, and −5.69 Kcal/mol, respectively (Table 3). Donepezil, a commonly used medicine, was found to interact with residues TYR 465, TYR 124, HIS 447, and SER 203 in the AChE binding site. In addition to GLU 202 and SER 203, the GLY 121 and GLY 122 residues on AChE were anticipated to be binding sites for gallic acid. Acetylcholine, on the other hand, was predicted to bind to a number of different residues on AChE, particularly SER 203, GLY 121, GLY 122, ALA 204, ARG 296, and PHE 295 (Table 3 and Figure 1).

### 2.4. In Vivo Anti Alzheimer Potentials

After 6 weeks of treatment, the rats’ body weights were measured, the body weight gain was significantly more decreased in the induction group than the control group. Moreover, the body weight remained significantly less than the control group after treatment with gallic acid and OPE (Table 4).

#### 2.4.1. Effect of Donepezil, Gallic Acid and OPE Treatment on Brain Aβ42, Total Cholesterol and Phospholipids Levels

Brain Aβ42 levels (pg/g tissue) were measured by sandwich ELISA. Aluminum chloride injection at a dose of 70 mg/kg significantly increased the level of Aβ_42_ 3.60-fold comparing to the control (19.18 ± 1.15 vs. 5.32 ± 0.98). Oral administration of donepezil (2.25 mg/kg), gallic acid (50 mg/kg), OPE (100 mg/kg), and OPE (200 mg/kg) before aluminum chloride injection significantly reduced the level of Aβ_42_ to 7.97 ± 0.63, 13.86 ± 1.70, 16.73 ± 0.97, and 11.55 ± 1.02 pg/g tissue, respectively at (*p* < 0.05) (Table 4).

The brain’s total cholesterol (TC) and phospholipid (Ph. Lipid) levels (mg/g protein) and TC/phospholipid ratio analysis showed that brain TC levels were significantly elevated by a factor of two in the aluminum chloride group. However, phospholipid levels decreased from 72.33 ± 1.19 mg/g of protein in the control group to 33.50 ± 0.86 mg/g protein in the AD-induced group. Moreover, aluminum chloride significantly increased the TC/phospholipid ratio 4.5-fold as compared to control (Table 4). In donepezil-, gallic acid-, OPE 100-, and OPE 200-treated groups, the brain TC levels were 70.65 ± 1.72, 51.43 ± 1.46, 58.83 ± 1.04, and 44.45 ± 0.90 (mg/mg protein), respectively. There was no significant different in brain phospholipid level and TC/phospholipid ratio between the OPE 200-treated group and the control group.

#### 2.4.2. Effect of Donepezil, Gallic Acid, and OPE Treatment on Oxidative Stress Parameters in Brain

Particularly in rat brain tissue, lipid peroxidation (LPO) was evaluated to assess the oxidative stress. Thiobarbituric acid-reactive substances (TBARS) (umol/g × 10^−3^) measurement in brain (Table 4) showed that aluminum chloride injection increased TBARS levels more significantly than the control group at 5.43 ± 0.83 and 1.32 ± 0.15, respectively, at (*p* < 0.05), while their levels in either donepezil-, gallic acid-, OPE 100-, or OPE 200-treated groups were significantly decreased to 3.67 ± 0.59, 2.41 ± 0.21, 4.25 ± 0.38, and 2.34 ± 0.22, respectively, in the brain than that of the aluminum chloride-treated rats. The NO level (µm/mg protein) in the brain (Table 4) was 12.43 ± 0.96 in control group. After treatment with aluminum chloride, the brain NO levels increased significantly to 32.76 ± 1.47 compared to the control. In donepezil-, gallic acid-, OPE 100-, and OPE 200-treated groups, the NO levels decreased significantly (*p*< 0.05) to 23.35 ± 1.31, 23.54 ± 0.73, 22.58 ± 0.79, and 21.86 ± 1.68, respectively, compared to the aluminum chloride group. Brain GSH level (mg/mg protein) × 10^−2^ in brain (Table 4) significantly decreased at (*p* < 0.05) in the aluminum chloride-injected group (0.24 ± 0.12) as compared to the control group (0.43 ± 0.03). In donepezil-, gallic acid-, OPE 100,- and OPE 200-treated groups the brain GSH levels were increased significantly compared to the aluminum chloride group.

#### 2.4.3. Effect of Donepezil, Gallic Acid, and OPE Treatment on Brain Antioxidant Enzymes

**Figure 2:**
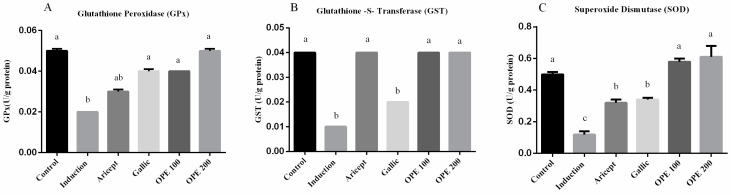
Effect of Aricept, gallic, OPE 100, and OPE 200 on brain antioxidant enzymes. Glutathione peroxidase (GPx) (**A**), Glutathione-S-transferase (GST) (**B**) and superoxide dismutase (SOD) (**C**) activities. Values represent the mean ± SD of six rats. ANOVA (one-way) followed by Tukey’s test. Values bearing the same letters showed no significant difference (*p* < 0.05). The results are sorted in descending order: a < b < c.

Brain GPx, GST and SOD activities (U/g protein) were measured and shown in (Figure 2A–C, respectively). The administration of aluminum chloride resulted in a significant decrease in GPx, GST, and SOD activities in brain at *p* < 0.05, comparing to their corresponding values in the control group. Daily administration of either donepezil, gallic acid, OPE 100, and OPE 200 with aluminum chloride injection resulted in a significant increase in the activities of GPx, GST, and SOD in brain tissue (GPx: 1.5-, 2-, 2-, and 2.5-fold, respectively); (GST: 4-, 2-, 4-, and 4-fold, respectively); (SOD: 2.7-, 2.8-, 4.8-, and 5.1-fold, respectively), comparing to the aluminum chloride group (induced group).

#### 2.4.4. Effect of Donepezil, Gallic Acid, and OPE Treatment on Brain AChE Activity and Gene Expression Levels (Hypothalamus)

**Figure 3:**
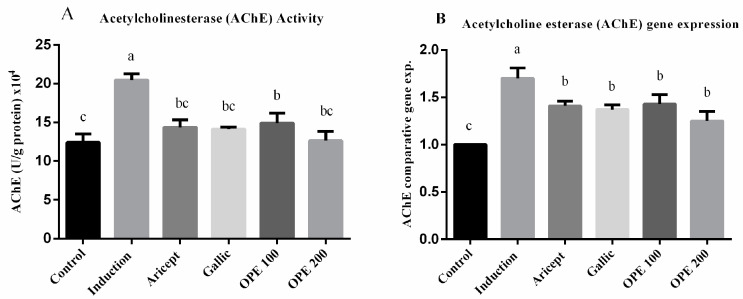
Effect of Aricept, gallic, OPE treatment on brain AChE activity (**A**) and gene expression levels (hypothalamus) (**B**). Values represent the mean ± SD of six rats. ANOVA (one-way) followed by Tukey’s test. Values bearing the same letters showed no significant difference (*p* < 0.05). The results are sorted in descending order: a < b < c.

AChE activity (U/g protein) × 10^4^ was significantly rose (1.6-fold) in the aluminum chloride treated group as compared to the control group (20.46 ± 0.81 vs. 12.4 ± 2.10) at *p* < 0.05 (Figure 3A). AChE activity in the brain tissue of aluminum chloride-treated rats that received either donepezil (14.36 ± 0.96), gallic acid (14.13 ± 0.25), OPE 100 (14.90 ± 1.30), or OPE 200 (12.64 ± 1.20) was significantly less than that of the aluminum chloride-treated rats (*p* < 0.05). Daily administration of donepezil (2.25 mg/kg) significantly decreased brain AChE activity as compared to the aluminum chloride group. It also showed a non-significant change in brain AChE activity compared to the control. The mRNA levels of AChE were measured using qRT-PCR analysis and the results are shown in (Figure 3B). The results showed that daily intraperitoneal injection of aluminum chloride for 6 weeks significantly upregulated the mRNA expression level of AChE as compared with the control group (1.70 ± 0.11 vs. 1.000 ± 0.000). Animals administered donepezil, gallic acid, OPE 100, and OPE 200 registered a significant decrease in the mRNA expression levels of AChE as compared with the aluminum chloride-treated group. Gallic acid-, OPE 100-, and OPE 200-treated groups showed a non-significance in AChE gene expression level compared to the donepezil-treated group (*p* < 0.05).

#### 2.4.5. Brain (Hippocampus) Gene Expression

**Figure 4:**
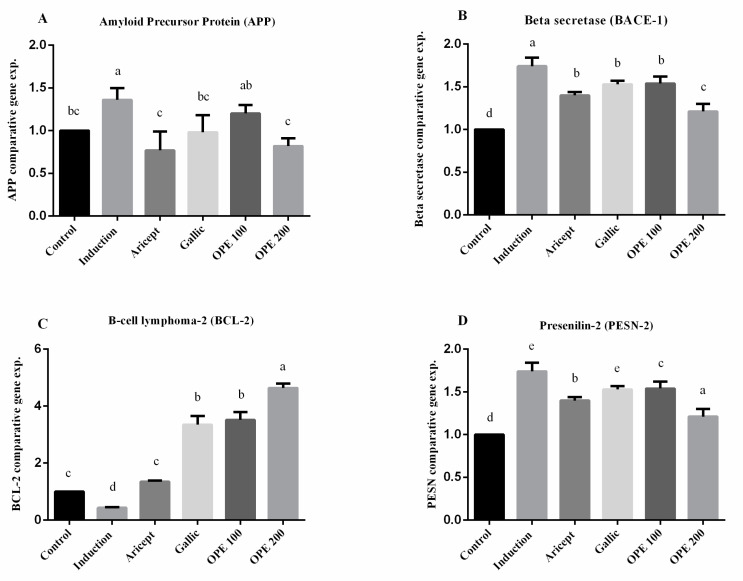
Effect of Aricept, gallic, OPE 100, and OPE 200 on brain (hippocampus) gene expression of amyloid precursor protein (APP) (**A**), beta secretase (BACE-1) (**B**), B-cell lymphoma-2 (BCL-2) (**C**) and presenilin-2 (PSEN-2) (**D**). Values represent the mean ± SD of six rats. ANOVA (one-way) followed by Tukey’s test. Values bearing the same letters showed no significant difference (*p* < 0.05). The results are sorted in descending order: a < b < c < d < e.

Brain (hippocampus) gene expression showed that intraperitoneal injection (ip) injection of aluminum chloride for 6 weeks significantly upregulated the mRNA expression level of APP as compared with the control group (1.36 ± 0.14 vs. 1.000 ± 0.000). Animals administered donepezil, gallic acid, OPE 100, and OPE 200 exhibited a significant decrease in the mRNA expression levels of APP as compared with the aluminum chloride-treated group. Results reveal that there was no significance in mRNA expression level of APP between the gallic acid-treated group and the control group (0.98 ± 0.20 vs. 1.000 ± 0.000). Additionally, OPE 200 treatment showed significant decrease in APP gene expression compared to the control group (0.82 ± 0.09 vs. 1.000 ± 0.000) (*p* < 0.05) (Figure 4A). Considering the beta secretase (BACE-1) gene, the results showed that intraperitoneal injection (ip) injection of aluminum chloride significantly upregulated the mRNA expression level of BACE-1 as compared with the control group (1.74 ± 0.10 vs. 1.000 ± 0.000). Animals administered donepezil, gallic acid, OPE 100, and OPE 200 represented a significant decrease in the mRNA expression levels of BACE-1 as compared with the aluminum chloride-treated group. Moreover, there was no significant difference in BACE-1 gene expression in donepezil-, gallic acid-, and OPE 100-treated groups (1.40 ± 0.04, 1.53 ± 0.04, 1.54 ± 0.08), respectively, at (*p* < 0.05) (Figure 4B). However, in the case of BCL-2 gene expression, results showed that daily ip of aluminum chloride significantly downregulated the mRNA expression level of BCL-2 as compared with the control group (0.43 ± 0.02 vs. 1.000 ± 0.000). Animals administered donepezil, gallic acid, OPE 100, and OPE 200 registered a significant increase in the mRNA expression levels of BCL-2 as compared with the aluminum chloride treated group. Gallic acid-, OPE 100-, and OPE 200-treated groups showed a significant increase in BCL-2 gene expression level compared to the donepezil-treated group (*p* < 0.05) (Figure 4C). Gene expression analysis of PSEN-2 showed that daily intraperitoneal injection of aluminum chloride significantly downregulated the mRNA expression level of PSEN-2 as compared with the control group (0.55 ± 0.16 vs. 1.000 ± 0.000) (Figure 3). Animals administered donepezil, OPE 100, and OPE 200 registered a significant increase in the mRNA expression levels of PSEN-2 as compared with the aluminum chloride-treated group. The gallic acid-treated group showed a non-significant change in PSEN-2 gene expression level compared to the induced group (*p* < 0.05). At the same time, OPE 100 and OPE 200 showed a more significant increase in PSEN-2 levels than the control group at (*p* < 0.05).

### 2.5. Electron Microscope Observations

**Figure 5:**
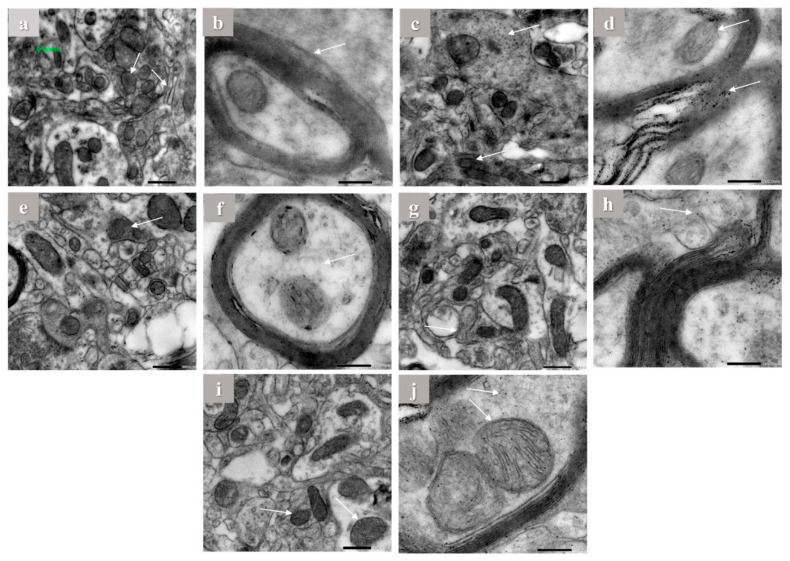
The electron microscope imaging of the brain hippocampus of Aricept-, gallic-, caffeine-, OPE 100-, and OPE 200-treated groups. Normal control group showed normal Golgi apparatus architecture without dilation (**a**,**b**), induced group showed amyloid beta deposition in myelin sheath (**c**,**d**), Aricept protective group showed concave synapse curvature and no amyloid beta deposition appeared (**e**,**f**), OPE 200 protective group showed asymmetric synapses with concave curvature and no amyloid beta deposition (**g**,**h**), and gallic acid protective group showed Golgi apparatus showed less dilation than induced group (**i**,**j**).

Hippocampus of brain tissues of control, aluminum chloride, and treated groups were examined using electron microscope by the end of the experiment. The hippocampus of control rats showed normal mitochondrial configuration, Golgi apparatus, and asymmetric concave synapses without any abnormal structures as shown in (Figure 5a,b). On the other hand, it is clearly visible that aluminum chloride group showed more abnormal structures and organelles compared to other groups. This is indicated by flat and convex synapses, a concentric configuration of mitochondrial cristae, dilation of Golgi apparatus, and extra amyloid beta deposition compared to the normal group (Figure 5c,d). The donepezil-treated group showed concave synapses curvature and no more amyloid beta deposition appeared (Figure 5e,f). Furthermore, the gallic acid-treated group showed asymmetric synapses with concave curvature and slight amyloid beta deposition (Figure 5g,h). In addition, in the OPE 200-treated group, Golgi apparatus showed less dilation than in the induced group, less amyloid beta deposition and the treated group was mostly near to the control group, which showed some protective effect against aluminum chloride-induced abnormal structures as shown in (Figure 5i,j), which are consistent with the biochemical marker profile. Considering mitochondria length and width, the results showed that daily intraperitoneal injection of aluminum chloride significantly decreased the mitochondrial length from 435.67 ± 1.24 to 186.30 ± 0.65 nm and the mitochondrial width from 261 ± 1.00 to 157 ± 2 in control group comparing to the induced group (Table 5). Animals administered donepezil registered a significant increase in mitochondrial length and width as compared with the aluminum chloride treated group. Moreover, animal groups administered gallic acid and OPE 200 separately registered a significant change in mitochondrial length and width as compared with the donepezil-treated group (*p* < 0.05); the are results shown in (Table 5).

## 3. Discussion

### 3.1. Phytochemicals Content and HPLC Analysis

Polyphenols are bioactive plant secondary metabolites with many important functions. These include long-term protection from vascular illnesses, chemoprotective action, antioxidant, neuroprotective, and anti-inflammatory effects [24]. Numerous studies have been conducted, and more are planned, to determine the feasibility of recovering polyphenolic compounds from agri-food wastes as a high-value biorefining option [24]. Orange peel extract (OPE) contains a moderate concentration of polyphenols and flavonoids (Table 1); fractionation and identification of these polyphenols by HPLC reveals that the extract contains twelve different polyphenolic and flavonoid compounds ranged from 3388.60 to 47.08 (μg/100 g) for gallic acid and cinnamic acid, respectively (Table 2). The most prevalent polyphenol in OPE, gallic acid, was found to have beneficial effects in treating a variety of diseases and conditions, including those related to the gut, the brain, the metabolism, and the heart [15]. In addition, it is a known antioxidant substance with neuroprotective activities in several types of neurodegeneration, neurotoxicity, and oxidative stress [25]. Another polyphenol found in high concentrations in OPE, rutin, protects neurons from the neurodegenerative consequences of prion buildup by boosting neurotropic factor synthesis and blocking apoptotic pathway activation in neural cells [26]. In regard to naringenin, another polyphenol containing OPE, it has demonstrated a wide variety of therapeutic effects, primarily due to its antioxidant activity. They have a direct impact on scavenging free radicals, and they also interact with various signal transduction pathways involved in the redox response, which leads to an increase in the internal antioxidant defense system and promotes neuroprotection [27]. Additionally, ferulic acid has been shown to have neuroprotective benefits in Parkinson’s disease cell models by inducing autophagy [28]. Moreover, quercetin possess neuroprotective action by acting with different mechanisms on the microglial cells of CNS and influences microRNA expression in order to regulate the inflammation, differentiation, proliferation, apoptosis, and immune responses [29]. Many other polyphenols and flavonoids were present in OPE with different biological activity. The interactions between these bioactive compounds may greatly improve their therapeutic effects and reduce their negative effects [30]. It has been found that the pharmacological effects of many commonly used herbs are significantly enhanced when administered together [31]. Thus, using the OPE as a mixture of bioactive compounds was interesting and may display a new properties added for each individual compounds.

### 3.2. Characterization of OPE

Antioxidant and anti-acetylcholinesterase activities were investigated as a first step to evaluate the biological activities of OPE. The ABTS, DPPH, and hydroxyl radical scavenging assays are significant in vitro analyses for studying OPE’s antioxidant efficacy (Table 1). Both techniques (ABTS and DPPH) use decolorization assays to identify antioxidants that prevent the formation of ABTS radical cations and DPPH radicals [32]. Because both techniques result in the same chemical characteristic of H or electron donation to the antioxidant, ABTS activity was closely associated with DPPH in the majority of experiments to measure antioxidant properties [33]. Hydroxyl radicals are very reactive and transient [34]. Because of their extreme reactivity, hydroxyl radicals cause tremendous harm to cells and their individual components, which then affects organisms as a whole [35]. It has previously been shown that a number of flavonoids can scavenge OH radicals [36]. Since free radicals have a detrimental role in Alzheimer’s disease progression, radical scavenging activities are crucial [37]. The present study indicates the half maximal inhibitory concentration (IC_50_) values denoted the concentration of OPE required to scavenge 50% of free radicals. OPE’s potential efficacy in combating free radical-induced diseases is likely attributable to the presence of a number of phenolic compounds and flavonoids. According to previous results with donepezil, the standard AD treatment exhibited IC_50_ 133 ± 4.5 μg/mL using DPPH assay [23]. In this study, OPE showed IC_50_ 67.90 ± 1.05 μg/mL. Thus, OPE has antioxidant activity, which is absent in donepezil. The previous antioxidant result of OPE is promising, suggesting that OPE can be used as a natural antioxidant. The effectiveness of OPE and gallic acid in inhibiting AChE, an enzyme that is hyperactivated in AD, was also investigated in vitro to compare with the standard AChE inhibitor, donepezil. The inhibitory concentrations (IC_50_) of OPE and gallic acid were 0.87 ± 0.025 and 2.45± 0.001 (mg/mL), respectively (Table 1). Results reveal that the inhibitory effect of OPE did not depend on the presence of gallic acid only but the presence of gallic acid in combination with other compounds such as polyphenols or flavonoids. OPE contain ellagic acid (EA) at the concentration of 336.16 (μg/100 g), which may contribute the anti-acetylcholinesterase activity of the extract. According to another study, artichoke waste extract was found to have an IC_50_ for AChE inhibition of 5.705 ± 0.0157 mg/mL; this may be because of ellagic acid contents [23]. In addition, the presence of gallic acid and ellagic acid in the methanolic extract of *Terminalia chebula* resulted in considerable cholinesterase inhibition (IC_50_ = 180 ± 14.6 mg/mL) [38]. In further addition, ellagic acid inhibited AChE in an animal model of Alzheimer’s disease [39]. According to our results, OPE might express a good result in treating Alzheimer disease model by its potential antioxidant and anti-acetylcholinesterase activity.

One way to screen for drugs that will work on a certain biological target is by computational docking, in which test ligands are placed in a docking simulation and their binding mode to the target is predicted [40]. Variable hydrogen binding sites were predicted using in silico docking investigations of donepezil, gallic acid, and acetylcholine against human acetylcholinesterase (PDB code: 4EY7) (Table 3; Figure 1). These findings show that gallic acid, when compared to the native substrate (acetylcholine) and donepezil, exhibited a lower binding energy. More research is needed to confirm that OPE, rather than its individual polyphenols, has anti-AChE activity in vivo.

### 3.3. The Possible Protective Effect of OPE against AD Rat Model Induced by Aluminum Chloride Injection

Aluminum chloride injection produced severe decline in cognitive capabilities and related symptoms of neurodegeneration in the brain. Furthermore, altered mitochondrial function presented, which might underlie neuronal dysfunction in AD [41]. In the current study, daily i.p. injection of aluminum chloride for 6 weeks produced severe decline in cognitive capabilities and related symptoms of neurodegeneration in the brain, including increased both gene expression and activity of AChE and Aβ42 levels. Aluminum chloride administration also caused oxidative damage (elevation in TBARS, NO, and decrease of reduced GSH level and reduce activity of the antioxidant enzyme). Furthermore, aluminum chloride produced downregulation of the amyloidogenic-related genes PSEN-2, BCL2, and upregulation of APP and BACE-1 gene expressions. Aluminum is a cholinotoxin, according to the literature, because it disrupts the cholinergic system [42]. It has been reported that aluminum chloride cholinotoxin activity in rats induces senile dementia of Alzheimer’s in animal models [43]. Its neurotoxic effects could be explained by many reported mechanisms, such as Aβ deposition in neurons. Additionally, stimulating AChE activity in the neuritic plaques and neurofibrillary tangles around regions [44]. Aluminum chloride injection at dose of 70 mg/kg for 6 weeks exerts significant effect on biochemical and behavioral than the dose of 50 mg/kg [43]. Thus, the present dose of aluminum chloride used was 70 mg/kg daily for 6 weeks. It has been reported that gallic acid (GA) has a protective role in Alzheimer’s disease (AD) and neurodegeneration at dosages equivalent to 20 mg/kg bw [45]. Antioxidant [46] and neuroprotective properties of purified GA were reported in multiple scientific studies [47,48]. The present study is the first to deal with OPE as a source of gallic acid with other compounds mixed that may play a role in AD disease.

### 3.4. Effect of Donepezil, Gallic Acid, and OPE Treatment on Brain Aβ42 Level, TC, and Phospholipids in the Brain

The amyloid cascade hypothesis proposed that the key clinical hallmarks of Alzheimer’s disease were extracellular deposits of A peptides as senile plaques, intraneuronal neurofibrillary tangles (NFTs), and extensive neuronal death [49,50]. Therefore, Aβ peptides have dominated new therapeutic research over the past twenty years as a possible target for AD [51]. Thus, Aβ42 level in rat’s brain was an important marker to evaluate [52]. Aluminum chloride injection for 6 weeks resulted in a significant 3.6-fold increase in Aβ42 level in rat’s brain compared to control (Table 4). Aβ42 has been reported to bind with the membrane negatively charged phospholipids, leading to conformational change of other nearby lipids leading to membrane disruption and cell death [53]. On the other hand, oral administration of OPE (200 mg/kg) before aluminum chloride injection significantly reduced the level of Aβ42 by 40.04% compared to those of the aluminum chloride-treated group (*p* < 0.05). In the case of administration of gallic acid at the concentration of 50 mg/kg the Aβ42 deposition significantly decreased compared to the aluminum chloride-treated group by 27.73%. Gallic acid is one of many chemicals found in tea extracts that have been demonstrated to have anti-amyloid characteristics, and it has been linked to the positive health effects [54]. This common polyphenol is thought to not only mediate oxidative stress and inflammation, but also to directly prevent the development of amyloid fibrils, both of which are major contributors to neurodegenerative disorders [55,56]. Many other detected polyphenols such as naringenin may influence the OPE results. Pretreatment with naringenin reduces Aβ-induced impairment of learning and memory by inhibiting lipid peroxidation and apoptosis, as shown by Ghofrani et al. [57]. One of the metabolites of curcuma is ferulic acid, which can be found in OPE. In addition to being able to scavenge reactive oxygen species, it has been shown to have neuroprotective properties by interacting directly with Aβ mature fibrils, which may lead to the disintegration of these aggregations [58,59]. Moreover, quercetin destabilizes preformed mature Aβ fibrils by strengthening the hydrophobic interaction between the phenyl rings and the β-sheet structures of Aβ [60,61].

Aluminum chloride injection increased the brain’s total cholesterol (TC) twofold and reduced the level of brain phospholipids to near half and also increased the TC/phospholipids ratio 4.71-fold as compared to control (Table 4). This adverse effect was reversed by oral intake of donepezil, gallic acid, and OPE. Oral administration of OPE restored the brain TC and phospholipids to near normal control values. The biochemical evidence is overwhelming that hypercholesterolemia is an important causal factor in the pathogenesis of AD. The biochemical evidence is overwhelming that hypercholesterolemia is an important causal factor in the pathogenesis of AD; moreover, cholesterol intracellularly influences Alzheimer’s disease APP synthesis and amyloid-beta formation [62]. A critical reason that Aβ aggregation is modulated by changes in cholesterol levels in the brain, which results in both β- and γ-secretase complexes located in cholesterol-rich lipid rafts of the plasma membrane in the brain; depleting cellular cholesterol as one of the therapeutic mechanism appears to inhibit BACE-1 enzyme [63]. Consistent with prior findings, aluminum chloride injection significantly increased Aβ42 levels through boosting cholesterol and lowering phospholipid levels in brain tissue of this generated model. Measuring of TC and phospholipid levels determine the mechanism of OPE and gallic acid protective mechanism against AD.

### 3.5. Effect of Donepezil, Gallic Acid, and OPE Treatment on Oxidative Stress Parameters in Brain

Many prooxidant markers were assessed in order to determine the effect of OPE and their polyphenol GA, regarding to TBARS, there were significant decreases in its levels in groups treated with donepezil, gallic acid, and OPE compared to the AD-induced group. There were no significant changes in TBARS levels between donepezil and OPE 100-treated groups. On the other hand, NO level showed significant decrease in groups treated with donepezil, gallic acid, and OPE compared to the AD-induced group (Table 4). The current results indicated that aluminum chloride increased the oxidative stress significantly (NO and TBARS) throughout the brain in agreement with Yuan et al. [64]. Groups treated with donepezil, gallic acid, and OPE exhibited a significant elevation in the antioxidant state in the rat’s brain via increase the level of GSH near to normal levels (Table 4). Aluminum chloride has also been found to disrupt metabolism, especially for antioxidants of low molecular weight such as glutathione, and thus exacerbate the rate of lipid peroxidation in the brain [65].

### 3.6. Effect of Donepezil, Gallic Acid, and OPE Treatment on Brain Antioxidant Enzymes

Results of brain antioxidant enzymes (GST, GPx, and SOD) in Figure 2 demonstrated that donepezil, gallic acid, and OPE treatment restores the antioxidant enzyme activity in normal levels near to the control values. It has been reviewed that in order to maintain normal neuronal function, reactive nitrogen species (RNS) and reactive oxygen species (ROS) are required [66]. Brain tissue is particularly vulnerable to oxidative stress due to high levels of oxygen (20% of the body’s oxygen), metals, polyunsaturated fatty acids and low antioxidant capacity [67]. So, improving the antioxidant state by administration of natural extract such as OPE could be helping to prevent the disease development or progression of AD.

### 3.7. Brain AChE Activity and Its Gene Expression Level on Brain (Hypothalamus)

In the present study, acetylcholinesterase activity and gene expression were investigated in brain hypothalamus (Figure 3). Aluminum chloride injection at 70 mg/kg increased AChE activity 1.65-fold over the control group and significantly upregulated the mRNA expression level of AChE as compared to the control group (1.706 ± 0.11 vs. 1.000 ± 0.17). The present data together with the Kaizer report indicated that aluminum chloride produced severe cholinergic neuronal dysfunction deficits as well as increased AChE activity and hypothalamic AChE expression, which could explain the brain neurodegeneration [68]; it may then evaluate the effects of oral administration of donepezil (2.5 mg/kg), gallic acid (50 mg/kg), OPE (100 mg/kg), and OPE (200 mg/kg) before aluminum chloride injection on AChE activity and correlate these activities with their anti-AD effect. A previous study reported that prolonged administration of donepezil (Aricept^®^, 1.5 mg/kg orally, twice daily for 21 days) inhibited AChE activity and increase the ACh concentration in rats’ brains [69]. The obtained results from the in vivo Alzheimer study, regarding AChE activity and AchE gene expression, explained the obtained results from the in vitro anti-acetylcholinesterase activity and docking studies.

### 3.8. Brain (Hippocampus) Gene Expression

Numerous human diseases are characterized by alterations in gene expression, which have been successfully exploited to anticipate molecular and cellular pathways linked with pathological processes [70]. In the brain, the single-pass transmembrane protein amyloid precursor protein (APP) is rapidly and digested by a number of successive proteases, including the intramembranous γ-secretase complex [71]. Alternative cleavage of the APP by β-secretase (BACE1) and the γ-secretase complex lead to production of Aβ species, e.g., Aβ38, Aβ40, and Aβ42 [72]. From all the previous facts, APP gene expression level was estimated in this study to follow the possible mechanism of OPE and its polyphenol gallic acid in neural action and brain protection; animal groups administered donepezil, gallic acid, and OPE registered a significant decrease in the mRNA expression levels of APP as compared with the aluminum chloride-treated group. The OPE 200-treated group showed nonsignificant APP gene expression level with the donepezil-treated group. In addition, OPE 200 expressed a significant decrease in APP gene expression level compared to the control group (*p* < 0.05) (Figure 4). Since β-secretase (BACE-1) cleavage is a bottleneck for Aβ42 production, it is a promising therapeutic target for the development of inhibitors that reduce Aβ42 [73]. The pharmaceutical industry has concentrated on BACE1 inhibitors as a therapeutic strategy for AD to block the first stage of amyloid development [74]. However, in clinical trials, BACE1 inhibitors have failed due to potential side effects or lack of beneficial cognitive outcomes [75]. In this study, results showed that aluminum chloride injection caused a significant increase in BACE1 gene expression level when compared to the control group (Figure 4). There were no significant differences in BACE1 gene expression level between donepezil (2.5 mg/kg), gallic acid (50 mg/kg), as well as OPE 100. However, oral treatment by OPE 200 exhibited a more significant decrease BACE1 gene expression than donepezil (2.5 mg/kg), gallic acid (50 mg/kg), as well as OPE 100 treated groups. From the above results, the treatment with gallic acid and OPE contribute to Aβ42 production through the modulation of amyloid precursor protein (APP) and the attenuation of Aβ42 generator enzyme (BACE1) gene expressions. Amyloid beta peptide induces neuronal apoptosis and contributes to the pathophysiology of AD. Key apoptosis proteins such as Bax, Bcl-2, and cytochrome c have been demonstrated to regulate the triggered apoptotic pathway involving mitochondria [76]. Our results showed that aluminum chloride induced apoptosis via down regulate of BCL-2 gene expression that in agreement with Justin et al. [77] who use aluminum chloride at dose of 100 mg/kg. mRNA expression levels of BCL-2 in donepezil-, gallic acid-, OPE 100-, and OPE 200-treated groups showed significant 1.34-, 3.35-, 3.52-, and 4.64-fold increases, respectively, comparing to the control group (*p* < 0.05) (Figure 4). The relationship between the increase in the BCL-2 gene expression levels of OPE- and gallic acid-treated groups were much higher compared to the donepezil-treated group within the used doses. These are considered good results in the treatment of Alzheimer’s disease, as evidenced by the APP and BACE1 gene expression results shown above. This early onset of the inherited form of Alzheimer’s disease has been linked to a lack of PSEN-2 function caused by mutation, which leads to incomplete digestion of the amyloid peptide and may contribute to increased vulnerability in the brain [78]. Oral administration of donepezil (2.5 mg/kg), gallic acid (50 mg/kg), OPE 100 (100 mg/kg), and OPE 200 (200 mg/kg) showed significant upregulation in the PSEN-2 gene expression comparing to the aluminum chloride-induced model (Figure 4). The findings in the present study are consistent with previous studies, which reported that quercetin doses (25 and 50 mg/kg) that were orally administered for 28 days inhibited the amyloidogenic pathway via suppression of APP, BACE1, APH1, and PSEN1 gene expression levels in the hippocampus [79]. From our previous results, the positive impact of OPE treatment on reversing aluminum-induced AD was confirmed through protein and gene analysis.

### 3.9. Electron Microscope

Neurodegenerative diseases have a vicious cycle of cell death caused by oxidative stress and mitochondrial malfunction [80]. Both Aβ42 and APP have been found to localize to mitochondrial membranes, where they are thought to interact with mitochondrial proteins, block the transport of nuclear-encoded mitochondrial proteins to mitochondria, disrupt the electron transport chain, increase production of reactive oxygen species, damage mitochondria, and ultimately impair neuronal function [81]. Recent studies demonstrated that altered mitochondrial function might underlie neuronal dysfunction in AD [82]. Accordingly, in order to investigate the mitochondrial ultrastructures and possible mechanical changes, electron microscopic studies were assessed. Administration of aluminum chloride for 6 weeks resulted in ultrastructure alterations in the hippocampus (Figure 5), such as rupture of the mitochondrial membrane and a concentric configuration of mitochondrial cristae. Synaptic loss is the best correlate of cognitive dysfunction in AD [83]. The present transmission electron microscopy showed morphological abnormalities including flat and convex curvatures of synapses. These findings are in line with the idea that mitochondrial damage in both the pre- and post-synaptic regions contributes to age-related synaptic loss. As the number of asymmetric synapses with concave curvature declined with age, the number of asymmetric synapses with flat and convex curvatures increased [84]. However, gallic acid was studied in vivo before at 100 mg/kg bw [15]. However, gallic acid in OPE acts perfectly compared to pure gallic acid. By analyzing the in silico, in vitro, and in vivo results, we can conclude that crude OPE can used as a treatment for Alzheimer’s more effectively than gallic acid and donepezil alone.

## 4. Material and Methods

### 4.1. Plant Materials

Orange fruits were collected from an Egyptian market in April 2019. Clean orange peels were dried on a well-ventilated burner at 50 degrees Celsius, then ground into a powder using a mechanical grinder and frozen at −20 degrees Celsius for later use.

### 4.2. Preparation of Orange Peel Extract

#### Conventional Extraction (CE)

Approximately 20 g of dried powdered orange peel was added to 200 mL of distilled water and left to ferment at 37 degrees Celsius for three days. According to Awad et al. [85], the extract was filtered, condensed using a rotary machine, lyophilized using a freeze-dryer, and then stored at −20 °C until further analysis was performed.

### 4.3. Phytochemical Analysis

#### 4.3.1. Total Phenolic Content (TPC)

Using the Folin–Ciocalteu spectrophotometric method, the total phenolic content (TPC) of orange peels extract was calculated, and the results were presented as microgram gallic acid equivalents (GAE) per milligram (mg) of dry extract [86].

#### 4.3.2. Total Flavonoid Assay (TFC)

The aluminum chloride colorimetric method was used to determine the total flavonoid concentration [87]. The total flavonoid concentration was reported as μg quercetin (Q) equivalent per milligram (mg) of dry extract

### 4.4. HPLC Polyphenol Identification of Orange Peels Extract

Orange peel extract was subjected to HPLC for identification of its polyphenols and flavonoids, according to Tomaino et al. [88], using an Agilent 1260 series. Results for the major phenolic components were reported in units of micrograms per 100 g of extract.

### 4.5. Characterization of Orange Peels Extract

#### 4.5.1. Antioxidant Activity

The antioxidant activity of the OPE was assayed using DPPH, ABTS, and hydroxyl radical scavenging assays. The DPPH% free-radical assay was carried out according to Bandoniene et al. [89]. For the ABTS method, the ability to scavenge free ABTS radicals was applied based on a published protocol Re et al. [90]. Scavenging tests for hydroxyl radicals were performed using the Fenton reaction according to Ozyürek et al. [91].

#### 4.5.2. Anticholinesterase Potentials

In vitro anticholinesterase potentials of citrus peel extract and gallic acid was carried out according to Abd El-Aziz et al. [23]. All the reactions were performed in six replicates.

### 4.6. Molecular Docking Studies

The most common polyphenol through HPLC analysis were further subjected to molecular docking. Donepezil served as the reference standard. All docked ligands were drawn as 3D structures in ACD/ChemSketch. The ligand was docked into AChE’s stable binding pocket. Protein Data Bank provided the 3D structure of AchE (PDB code: 4EY7) [92] and molecular docking studies were performed using AutoDockTools-1.5.6rc3, Cygwin 64 Terminal, and Chimera 1.8.1.

### 4.7. In Vivo Experiment of OPE Anti-Alzheimer Potentials

#### 4.7.1. Experimental Design

Thirty-six mature male albino rats (weighing 100–110 g) were obtained from the National Research Center in Alexandria (Egypt). To develop learning and memory deficits, male albino rats were intraperitoneally (ip) injected at 70 mg/kg body weight with aluminum chloride (AlCl_3_·6H_2_O), dissolved in physiological saline solution, once a day for 6 weeks [93]. Normal animals separately received saline as vehicles. The rats were randomly divided into six groups as follows:

Control group (n = 6) received saline solution (0.5 mL/day, i.p) for only 6 weeks. Induced group (n = 6) received AlCl_3_·6H_2_O (70 mg/kg, ip) dissolved in saline for 6 weeks. Gallic acid protective group (n = 6) received gallic acid (50 mg/kg, oral) dissolved in normal saline, followed by AlCl_3_·6H_2_O (70 mg/kg, ip) after 60 min for 6 weeks [94]. Orange peel protective group (n = 6) received OPE (100 mg/kg, oral) dissolved in normal saline, followed by AlCl_3_·6H_2_O (70 mg/kg, ip) after 60 min for 6 weeks. Orange peel protective group (n = 6) received OPE (200 mg/kg, oral) dissolved in normal saline, followed by AlCl_3_·6H_2_O (70 mg/kg, ip) after 60 min for 6 weeks. Donepezil (Aricept)-treated group (n = 6) received Donepezil (2.25 mg/kg, oral) dissolved in saline, followed by AlCl_3_·6H_2_O (70 mg/kg, ip) after 60 min. Donepezil, a centrally acting cholinesterase inhibitor, was used as a positive control. Donepezil hydrochloride was obtained as 10 mg tablets and administered in doses of 2.25 mg/kg based on previous study of Haug et al. [95] (Aricept ^®^, Pfizer Egypt, S.A.E. Cairo, A.R.E. Under authority of Pfizer INC. USA).

After six-week treatment, rats were subjected for body weight then scarified. Brain tissue was homogenized at 10% 0.1 M phosphate buffer saline (PBS) at a pH of 7.4, and the mixture was centrifuged at 10,000× *g* for 20 min at 4 degrees Celsius. Protein level was measured by the method of Bradford et al. [96] for the supernatant. The standard was 1 mg/mL of bovine serum albumin (BSA). Furthermore, samples from brain hypothalamus and hippocampus washed by normal saline and stored in −80 °C for total RNA isolation and quantitative RT-PCR analysis. Electron microscope samples were prepared from brain hippocampal specimens according to Pejman et al. [97]. Briefly, semi-thin sections were made from the blocks. After toluidine blue observation, ultrathin brain slices (70–80 nm thick) were cut using a diamond knife, placed on 200 mesh copper grids, and stained for 15 min with 4% aqueous uranyl acetate, then for 8 min with Reynolds’ lead citrate. Transmission electron microscopy was used to examine the specimens (JEOL—JSM-1400 PLUS).

#### 4.7.2. Biochemical Parameters in Brain Homogenate

Malondialdehyde (MDA), a prominent byproduct of lipid peroxidation, was quantified spectrophotometrically by the thiobarbituric acid (TBA) reaction [98]. The level of Nitric oxide (NO) was determined using the Montgomery and Dymock [99] method. Reduced glutathione (GSH) content was determined as described previously [100]. The results of MDA, NO, and GSH were expressed as micromolars per gram of protein. The activity of Glutathione peroxidase (GPx) was measured as described previously [101]. The Glutathione S-transferases (GSTs) activity was determined as described previously [102]. The enzyme activity of GPx and GST were expressed as units per gram of protein. The activity of Superoxide dismutase (SOD) was assayed according to Marklund et al. [103]. The levels of total cholesterol and phospholipids were measured using an assay kit (Biosystems S.A., Spain and Bio-diagnostics, Egypt) (Biosystems S.A., Spain and Bio-diagnostic, Egypt) according to the manufacturer’s protocols. Acetylcholinesterase (AChE) activity was assayed as described by Ellman et al. [104]. The amount of acetylthiocholine iodide in micromoles hydrolyzed per minute per milligram of protein was used as one unit of AChE activity. AChE specific activity is measured in terms of units per gram of protein. The amyloid beta peptide 42 (Aβ42) level was measured in brain homogenate using the quantitative sandwich enzyme immunoassay technique (rat Aβ peptide 1–42 ELISA Kit CUSABIO, China) according to the manufacturer’s instructions.

#### 4.7.3. Hippocampal and Hypothalamic Tissue Separation and Total RNA Purification for qRT-PCR Analysis

Each rat was decapitated, and then their hippocampi and hypothalamuses were swiftly removed and frozen at −80 degrees Celsius for later use. Single-step RNA isolation using acid guanidinium thiocyanate–phenol–chloroform extraction was used for the manual extraction of RNA [105]. In order to perform quantitative real-time PCR, synthesized cDNA was combined with Power SYBR Green PCR Master Mix (Applied Biosystems) and a gene-specific primer (Bioneer, Daejeon, Korea), as seen in Table 6. Different real-time PCR reactions were performed for the target and GPDH (Rotor—Gene Q, QIAGEN Hilden, Germany). The conditions used were as follows: A denaturation step at 95 °C for 10 min was followed by 40 cycles of annealing at Tm [°C] for 15 s, followed by an extension step at 72 °C for 60 s. Step One Software calculated the detection threshold cycle number (Life Technologies). Each target’s mRNA expression was normalized to the expression of the GPDH gene and expressed as a fold change from the controls. There were three technical replicates for each sample. Using the the 2^−∆∆Ct^ technique, the fold changes in gene expression were measured [106].

### 4.8. Statistical Analysis of the Data

IBM SPSS version 16.0 was used for data analysis, which involved entering the data into a computer and processing it [107]. We used mean and standard deviation to characterize the quantitative data. The F-test (ANOVA) and Post Hoc test (Tukey) were used to compare the groups, while the paired *t*-test was employed to examine the two paired data. All results were considered statistically significant at the 5% level [108].

### 4.9. Ethical Approval

The procedures and protocols were used in the present research were approved by the Ethical Committee of Alexandria University (Protocol approval number: AU 04190727201). Animals were housed in a well-ventilated room on wood shavings polypropylene animal cages, four per cage, with free access to water and a standard chow diet. They were kept under standard environmental conditions of 22–25 °C, 12 h light/dark cycle. All the animals’ procedures and care were performed in accordance with the *Guide for the Care and Use of Laboratory Animals* [8].

## 5. Conclusions

It is commonly known that nutrition has a crucial influence in the onset and progression of non-communicable diseases. Orange peel extract (OPE) has many applications in food and disease treatment. In this study, in vitro and in silico analysis of OPE and derived gallic acid toward acetylcholinesterase showed an interested result. Moreover, OPE protects brain tissue by decreasing oxidative stress. The anti-Alzheimer’s disease properties of OPE include the inhibition of brain acetylcholinesterase activity and gene expression. Moreover, there was evidence of a decrease in Aβ42 levels and APP, BACE1 gene expression, and, furthermore, an increase in BCL-2 and PSEN-2 gene expression. Orange peel extract treatment against an aluminum chloride-induced AD model exhibited a promising results in the level of measured biochemical, molecular, and structural observations. Therefore, OPE may be formulated in certain kinds of dietary supplements, which may help in case of AD and other neurodegenerative disorders.

## Figures and Tables

**Table 1:** Orange peel extract (OPE) total phenolic, total flavonoids, and its functional properties.

Parameters	Results
TP	255.86 ± 1.77
TF	52.06 ± 1.74
Antioxidant activity	
DPPH	67.90 ± 1.05
ABTS	60.48 ± 0.91
Hydroxyl radical scavenging activity	63.70 ± 0.30
Anti-acetylcholinesterase activity	
OPE	0.87 ± 0.025 ^b^
Gallic acid	2.45± 0.001 ^c^

TP: total phenolics (mg of GAE/100 g); TF: total flavonoids (mg of catechol/100 g). For Antioxidant activity the IC_50_ value (μg/mL) of ascorbic acid was 2.33 ± 0.02. Regarding to anti-acetylcholinesterase activity the IC_50_ value (mg/mL) of Aricept was 0.0034 ± 0.0 ^a^. Results are expressed as means ± standard error of three measurements. Values bearing the same letters showed no significant difference (*p* < 0.05). The results are sorted in descending order: a < b < c.

**Table 2:** Phenolic compounds identified by high-performance liquid chromatography (HPLC) (µg/g) OPE.

Compounds	Chemical Name	Formula	R.T.	Conc. (μg/100 g)
**Gallic acid**	3,4,5-trihydroxybenzoic acid	C_7_H_6_O_5_	3.13	3388.60
**Chlorogenic acid**	(1S,3R,4R,5R)-3-[(E)-3-(3,4-dihydroxyphenyl)prop-2-enoyl]oxy-1,4,5-trihydroxycyclohexane-1-carboxylic acid	C_16_H_18_O_9_	3.49	874.25
**Catechin**	(2R,3S)-2-(3,4-dihydroxyphenyl)-3,4-dihydro-2H-chromene-3,5,7-triol	C_15_H_14_O_6_	3.84	ND
**Caffeine**	1,3,7-trimethylpurine-2,6-dione, guaranine, methyltheobromine	C_8_H_10_N_4_O_2_	4.03	ND
**Coffeic acid**	(2E)-3-(3,4-dihydroxyphenyl) prop-2-enoic acid, 3,4-dihydroxy-trans-cinnamate	C_9_H_8_O_4_	4.97	ND
**Syringic acid**	4-hydroxy-3,5 dimethoxy benzoic acid, gallic acid 3,5-dimethyl ether	C_9_H_10_O_5_	5.37	330.91
**Rutin**	2-(3,4-dihydroxyphenyl)-5,7-dihydroxy-3-[(2S,3R,4S,5S,6R)-3,4,5-trihydroxy-6-[[(2R,3R,4R,5R,6S)-3,4,5-trihydroxy-6- methyloxan-2-yl] oxymethyl] oxan-2-yl] oxychromen-4-one	C_27_H_30_O_16_	5.70	1952.64
**Pyro catechol**	3-(methylaminomethyl)benzene-1,2-diol;hydrochloride	C_8_H_12_ClNO_2_	5.82	0.00
**Ellagic acid**	3,7,8-tetrahydroxy-[1]-benzopyrano[5,4,3,-cde]-[1]-benzopyran-5,10-dione	C_14_H_6_O_8_	6.83	336.16
**O-coumaric acid**	(E)-3-(2-hydroxy phenyl) prop-2-enoic acid, 2-hydroxycinnamic acid	C_9_H_8_O_3_	7.72	183.16
**Vanillin**	4-hydroxy-3-methoxybenzaldehyde vanillic aldehyde	C_8_H_8_O_3_	8.38	ND
**Ferulic acid**	(2E)-3-(4-hydroxy-3-methoxyphenyl) prop-2-enoic acid, 3-methoxy-4-hydroxycinnamic acid	C_10_H_10_O_4_	8.80	1335.96
**Naringenin**	5,7-dihydroxy-2-(4-hydroxyphenyl)-2,3-dihydrochromen-4-one	C_15_H_12_O_5_	9.43	1967.43
**Propyl gallate**	propyl 3,4,5-trihydroxybenzoate	C_10_H_12_O_5_	10.22	1212.96
**4′,7-Dihydroxy isoFlavone**	8-[(2S,3R,4S,5S,6R)-4,5-dihydroxy-6-(hydroxymethyl)-3-[(2S,3R,4S,5R)-3,4,5-trihydroxyoxan-2-yl]oxyoxan-2-yl]-7-hydroxy-3-(4-hydroxyphenyl)chromen-4-one	C_26_H_28_O_13_	10.49	277.49
**Querectin**	2-(3,4-dihydroxyphenyl)-3,5,7-trihydroxychromen-4-one;dihydrate	C₁₅H₁₀O₇	10.69	1266.97
**Cinnamic acid**	(2E)-3-phenylprop-2-enoic acid	C_9_H_8_O_2_	11.22	47.08

**Table 3:** Docking substrates with AChE (PDB: 4EY7) and predicted binding information.

Ligand Name	Molecular Formula	Molecular Weight (g/mol)	Binding Energy (ΔG, Kcal/mol)	Binding Sites
**Gallic acid**	C7H6O5	170.12	−3.72	GLU 202, SER 203, GLY 121, GLY 122
**Acetylcholine**	C7H16NO2	146.21	−5.69	SER 203, GLY 121, GLY 122, ALA 204, ARG 296, and PHE 295
**Aricept**	C24H29NO3	379.5	−9.47	TYR 465, TYR 124, HIS 447, and SER 203

**Table 4:** Effect of Aricept, gallic, OPE 100, and OPE 200 on body weight gain, brain structure, and other oxidative stress parameters in brain tissue of different experimental rat groups treated with aluminum chloride.

Exp. Groups	Control	Induction	Aricept Protective Group	Gallic Protective Group	OPE 100 Protective Group	OPE 200 Protective Group
**Body weight gain**	54.56 ± 2.05 ^a^	34.14 ± 2.05 ^c^	39.47 ± 1.13 ^b^	34.66 ± 1.28 ^c^	34.65 ± 1.88 ^c^	40.00 ± 1.80 ^b^
**Brain structure**						
**Aβ42** **(Pg/g tissue)**	5.32 ± 0.98 ^f^	19.18 ± 1.15 ^a^	7.97 ± 0.63 ^e^	13.86 ± 1.70 ^c^	16.73 ± 0.97 ^b^	11.55 ± 1.02 ^d^
**Total cholesterol** **(mg/mg protein)**	46.11 ± 0.93 ^e^	94.88 ± 0.83 ^a^	70.65 ± 1.72 ^b^	51.43 ± 1.46 ^d^	58.83 ± 1.04 ^c^	44.45 ± 0.90 ^e^
**Phospholipids levels (mg/mg protein)**	72.33 ± 1.19 ^a^	33.50 ± 0.86 ^e^	60.73 ± 1.55 ^c^	38.20 ± 1.25 ^d^	67.53 ± 1.50 ^b^	71.30 ± 1.41 ^a^
**Total cholesterol/** **phospholipids**	0.63 ± 0.00 ^e^	2.83 ± 0.07 ^a^	1.16 ± 0.02 ^c^	1.34 ± 0.08 ^b^	0.87 ± 0.03 ^d^	0.62 ± 0.005 ^e^
**Parameters of oxidative stress**						
**TBARS ** **(umol/g × 10^−3^)**	1.32 ± 0.15 ^d^	5.43 ± 0.83 ^a^	3.67 ± 0.59 ^b^	2.41 ± 0.21 ^c^	4.25 ± 0.38 ^b^	2.34 ± 0.22 ^c^
**NO level ** **(µm/mg protein)**	12.43 ± 0.96 ^c^	32.76 ± 1.47 ^a^	23.35 ± 1.31 ^b^	23.54 ± 0.73 ^b^	22.58 ± 0.79 ^b^	21.86 ± 1.68 ^b^
**GSH level ** **(mg/mg protein × 10^−2^)**	0.43 ± 0.03 ^a^	0.24 ± 0.12 ^b^	0.60 ± 0.10 ^a^	0.58 ± 0.09 ^a^	0.55 ± 0.04 ^a^	0.62 ± 0.16 ^a^

Values represent the mean ± SD of six rats. ANOVA (one-way) followed by Tukey’s test. Values bearing the same letters showed no significant difference (*p* < 0.05). The results are sorted in descending order: a < b < c < d < e < f.

**Table 5:** Effect of OPE on brain (hippocampus) mitochondrial length and width (nm) of different experimental rat groups treated with aluminum chloride.

Groups	Control	Induction	Aricept	Gallic Acid	OPE 200
**Mit. Length (nm)**	435.67 ± 1.24 ^b^	186.30 ± 0.65 ^e^	368.27 ± 0.75 ^d^	407 ± 1.00 ^c^	450.33 ± 0.35 ^a^
**Mit. Width (nm)**	261 ± 1.00 ^b^	157 ± 2 ^d^	248.87 ± 0.90 ^c^	263.66 ± 2.54 ^b^	300.78 ± 1.09 ^a^

Values bearing the same letters showed no significant difference (*p* < 0.05). The results are sorted in descending order: a < b < c < d < e.

**Table 6:** Primers nucleotides sequence used in this study.

Primer Name	Primer Sequence from 5′–3′	Anealing Temp. °C
**Acetylcholinesterase (AChE)**	R-CCACCGATCCTCTGGACGAG F-CGCTCCTGCTTGCTATAGTG	60
**B-cell lymphoma-2 (BCL-2)**	F-ATGTGTGTGGAGAGCGTCAACC R-TGAGCAGAGTCTTCAGAGACAGCC	63
**Presenilin-2 (PSEN-2)**	F-GAGCAGAGCCAA ATCAAA GG R-GGGAGA AAGAACAGCTCGTG	60
**B–site APP-cleaving enzyme (BACE-1)**	F-CGGGAGTGGTATTATGAAGTG R-AGGATGGTGATGCGGAAG	60
**Amyloid precursor protein (APP)**	F-AGAGGTCTACCCTGAACTGC R-ATCGCT TACAAACTCACCAAC	54.9
**Glycerol-3-phosphate dehydrogenase (GPDH)**	F-ATTGACCACTACCTGGGCAA R-GAGATACACTTCAACACTTTGACCT	60–65

## Data Availability

Data is contained within the article.
